# Assessing the length of hospital stay for patients with myasthenia gravis based on the data mining MARS approach

**DOI:** 10.3389/fneur.2023.1283214

**Published:** 2023-12-14

**Authors:** Che-Cheng Chang, Jiann-Horng Yeh, Hou-Chang Chiu, Tzu-Chi Liu, Yen-Ming Chen, Mao-Jhen Jhou, Chi-Jie Lu

**Affiliations:** ^1^Department of Neurology, Fu Jen Catholic University Hospital, Fu Jen Catholic University, New Taipei City, Taiwan; ^2^PhD Program in Nutrition and Food Science, Fu Jen Catholic University, New Taipei City, Taiwan; ^3^School of Medicine, Fu Jen Catholic University, New Taipei City, Taiwan; ^4^Department of Neurology, Shin Kong Wu Ho-Su Memorial Hospital, Taipei City, Taiwan; ^5^Department of Neurology, Kaohsiung Medical University, Kaohsiung, Taiwan; ^6^Department of Neurology, Taipei Medical University, Shuang-Ho Hospital, New Taipei City, Taiwan; ^7^Graduate Institute of Business Administration, Fu Jen Catholic University, New Taipei City, Taiwan; ^8^Artificial Intelligence Development Center, Fu Jen Catholic University, New Taipei City, Taiwan; ^9^Department of Information Management, Fu Jen Catholic University, New Taipei City, Taiwan

**Keywords:** myasthenia gravis, multivariate adaptive regression splines, data mining, prognosis, hospitalization, machine learning

## Abstract

Predicting the length of hospital stay for myasthenia gravis (MG) patients is challenging due to the complex pathogenesis, high clinical variability, and non-linear relationships between variables. Considering the management of MG during hospitalization, it is important to conduct a risk assessment to predict the length of hospital stay. The present study aimed to successfully predict the length of hospital stay for MG based on an expandable data mining technique, multivariate adaptive regression splines (MARS). Data from 196 MG patients' hospitalization were analyzed, and the MARS model was compared with classical multiple linear regression (MLR) and three other machine learning (ML) algorithms. The average hospital stay duration was 12.3 days. The MARS model, leveraging its ability to capture non-linearity, identified four significant factors: disease duration, age at admission, MGFA clinical classification, and daily prednisolone dose. Cut-off points and correlation curves were determined for these risk factors. The MARS model outperformed the MLR and the other ML methods (including least absolute shrinkage and selection operator MLR, classification and regression tree, and random forest) in assessing hospital stay length. This is the first study to utilize data mining methods to explore factors influencing hospital stay in patients with MG. The results highlight the effectiveness of the MARS model in identifying the cut-off points and correlation for risk factors associated with MG hospitalization. Furthermore, a MARS-based formula was developed as a practical tool to assist in the measurement of hospital stay, which can be feasibly supported as an extension of clinical risk assessment.

## 1 Introduction

Myasthenia gravis (MG) is a neuromuscular junction disorder in which antibodies attack the post-synaptic proteins, which can cause muscle weakness and fatigue during repeated muscle contraction (1). MG is an uncommon disease that affects 15–25 people per 100,000 people (2). Currently, the complication rates of MG are improved under good management, and the treatment of MG has been well documented (3). However, the relapse rate is still variable and the severity varies from person to person; approximately 38% of patients with MG had remission, and 10% are resistant to traditional immunotherapy and need hospitalization (4). Even with the use of multiple drugs, some patients have poor symptom control and occasionally require repeated or prolonged hospitalization (4). However, it is currently difficult to predict who will require a longer stay in hospital or estimate the length of hospitalization because of complex clinical variability.

Most previous studies investigating predictors of prognosis and risk factors for MG symptom deterioration have been based on linear or logistic regression (5–9). Multiple linear regression (MLR) is a classic method used in many medical studies (10). However, MLR has limitations when the data contain non-linear variables. Using traditional methods for risk prediction and outcomes measurement in autoimmune diseases (including MG) is difficult because of the long-term course and complex phenotype. In addition, traditional methods cannot solve the problem of collinearity or non-linear relationships between variables. Recently, data mining methods have been used as alternatives to traditional statistical methods in medical research (11–13). They can process different types of input data that fill a gap in learning from clinical experience with computers capable of recognizing disease patterns and detecting disease features (14–18). Machine learning (ML) is one of the data mining tools that can provide computers with the ability to learn from experience (19–22). Multivariate adaptive regression splines (MARS), which is a data mining method, is a non-linear and non-parametric regression algorithm that does not require the specification of a functional form in advance (23). The MARS method can use a series of piecewise regression splines to process the unknown functional form which makes it appropriate for modeling complex non-linear relationships (24, 25).

Currently ML algorithms have been widely applied in medicine as they can effectively extract potentially useful information from datasets (26–29). However, their methods are not broadly used in the clinical evaluation of MG. The present study aims to investigate the relationship between the risk factors and the length of hospital stays based on MARS. With MARS, we developed a decision process for screening clinical factors associated with the length of hospital stay and also to construct an explainable prediction model successfully. Our findings suggest that the MARS model can help to identify cut-off points for risk factors association with MG hospitalization. Furthermore, a MARS-based formula was designed as an assisting tool to help with measurement of hospital stay.

## 2 Materials and methods

### 2.1 Participant and study design

**Figure 1:**
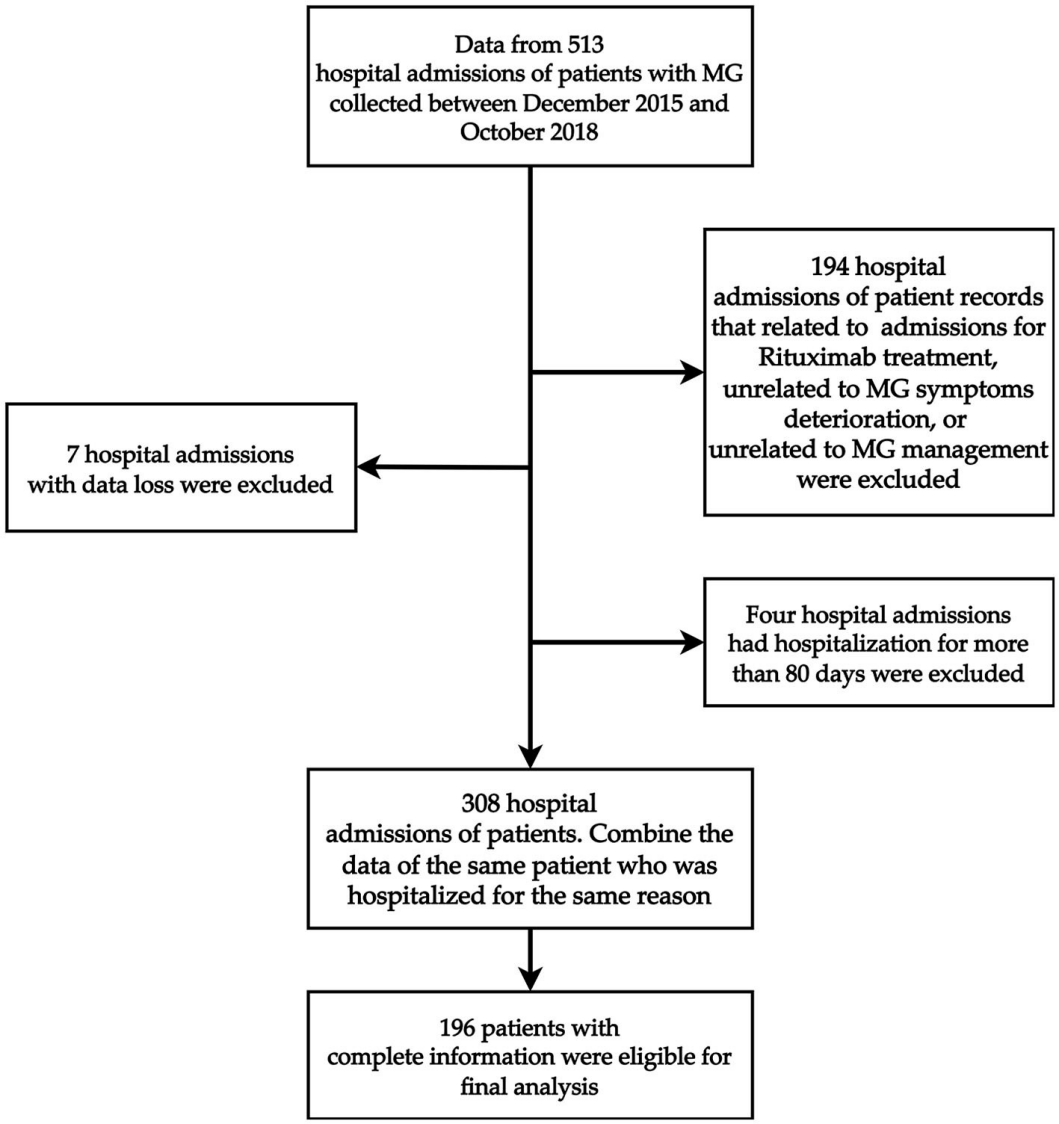
Overall flowchart of the participant enrollment process.

This retrospective study was performed from 513 hospital admissions of patients with MG at the Shin-Kong Wu Ho-Su Memorial Hospital in Taiwan between December 2015 and October 2018. Patients who were admitted for MG symptom deterioration or admission for MG-related management, including thymectomy or immunotherapy, were considered for enrollment. Furthermore, we considered as the outliers patients whose length of stay was greater than three standard deviations (SD) of the mean length of stay based on the raw data before filtration; therefore, we excluded four hospital admissions who had been hospitalized for more than 80 days. Figure 1 shows the detailed case identification process. After filtration, data from 196 patients were analyzed. Ultimately, a total of 196 patients were included in the analysis (Figure 1). The study protocol was evaluated and deemed acceptable by the Research Ethics Review Committee of the Shin Kong Wu Ho-Su Memorial Hospital (No. 20190109R). All of our methods were carried out in accordance with relevant guidelines and regulations.

The data of all patients were collected through a review of their admission medical records, and the detailed characteristics are shown in Table 1. Disease severity was graded according to the Myasthenia Gravis Foundation of America (MGFA) classification based on previous reviews that reported the clinical severity of the patient upon admission (30). A total of 19 clinical variables that may affect the length of hospital stay in patients with MG were assessed (6, 31, 32). Among them, average length of hospital stay was the target variable whereas the rest of the 18 variables were the predictor variables.

**Table 1:** Subject demographics.

**Characteristics**		**Value**	**Distribution in the dataset (** ***N*** **, %)**	
**Categorical variable**				
Sex		Male	77	39.3%
		Female	119	60.7%
MGFA clinical classification		I: Ocular muscle weakness	23	11.7%
		IIa: Mild limbs or axial weakness	22	11.2%
		IIb: Mild bulbar or respiratory weakness	49	25.0%
		IIIa: Moderate limbs or axial weakness	14	7.1%
		IIIb: Moderate bulbar or respiratory weakness	50	25.5%
		IVa: Severe limbs or axial weakness	0	0.0%
		IVb: Severe bulbar or respiratory weakness	25	12.8%
		V: Intubation	13	6.6%
Thymic histology	Thymoma	Yes	91	46.4%
		No	105	53.6%
	Hyperplasia	Yes	59	30.1%
		No	137	69.9%
Thymectomy		Yes	129	65.8%
		No	67	34.2%
Anti-AChR Ab		Yes	174	88.8%
		No	22	11.2%
Anti-MuSK Ab		Yes	8	4.0%
		No	188	95.9%
dSN		Yes	14	7.1%
		No	182	92.9%
AZA		Yes	71	36.2%
		No	125	63.8%
MMF		Yes	8	4.1%
		No	188	95.9%
OT		Yes	5	2.6%
		No	191	97.4%
IVIG		Yes	14	7.1%
		No	182	92.9%
PP		Yes	146	74.5%
		No	50	25.5%
IC		Yes	40	20.4%
		No	156	79.6%
RTX		Yes	3	1.5%
		No	193	98.5%
**Characteristics**		Value (Mean ± SD)	Distribution in the dataset (N)	
**Continuous variables**				
Age at admission (years)		49.4 ± 16.9	196	
Disease duration (Month)		72.7 ± 87.6	196	
Age at onset (years)		43.1 ± 17.4	196	
PSL Maximum daily dose (mg)		14.9 ± 16.3	196	
Average length of hospital stay		12.3 ± 9.0	196	

Hyperplasia, thymic hyperplasia; thymectomy, previous received thymectomy; Anti-AChR Ab, antibody against acetylcholine receptor; Anti-MuSK Ab, antibody against muscle-specific tyrosine kinase; dSN, double seronegative; AZA, treatment with azathioprine; MMF, treatment with mycophenolate; OT, treatment with tacrolimus; IVIG, treatment with intravenous immunoglobulin; PP, treatment with plasmapheresis; PSL, prednisolone; IC, treatment with intravenous corticosteroid; RTX, treatment with rituximab.

The definition of disease duration was from disease onset to the first visit after enrollment. The oral steroid dose before admission was recorded from the maximum dosages 1 month before hospitalization. The treatment during hospitalization, included plasmapheresis (PP), intravenous corticosteroid (IC), immunoglobulin (IVIG), and rituximab (RTX) administration was recorded. The serological status of MG autoantibodies included antibody against AChR, muscle-specific tyrosine kinase (MuSK), or double seronegative. We averaged the number of days spent by the same patient during different hospital stays, defined as the “average length of hospital stay.”

### 2.2 ML model of multivariate adaptive regression splines

MARS is a flexible procedure for finding variable interactions invented by Friedman (23). It can estimate non-linear data relationships by approximating with separate linear regression slopes in distinct intervals of the independent variable space (23–25). These lines, also known as splines, are piecewise linear lines that can best describe the data, whereas the points where the lines join together are the knots. These knots indicate each optimal cutting point of a variable from the data. Furthermore, the combination of splines and their corresponding knots are also known as the hinge functions, which take the form of max (0, *variable*−*knot*) or max(0, *knot*−*variable*). All hinge functions that describe a variable with their corresponding coefficients are known as basis functions (BF), in which each variable may have one or more BFs. Because each variable may have one or more BFs, they should be overall considered at the same time (23–25).

The building procedures for MARS involve several key steps. First, MARS starts by generating hinge functions to capture non-linear relationships in multivariate data. Second, to select the hinge functions that can form the most suitable BFs, a forward pass procedure is carried out by MARS. It will iteratively select and test the best BFs which considers both expansion and pruning via model selection criteria to optimize the model's complexity while minimizing prediction error. Then a backward pass procedure is conducted to simplify the model further by removing BFs with least contributions for making predictions. During this procedure, generalized cross-validation (a form of regularization that trades off goodness-of-fit against model complexity) is commonly utilized. MARS continuously refines and selects the most suitable BFs; the building procedures stop when further additions/eliminations of BFs do not significantly improve model performance. Hence, the final MARS equation is built and is composed of BFs from each selected variable.

The benefit of the MARS algorithm is that the estimated knots of important independent variables can provide useful information of the relationships between independent variables and the dependent variable, which helps to learn how non-linear features affect the target and select the important features. Thus, many studies from the clinical field utilize MARS because of the strengths that MARS can provide (33–37).

### 2.3 Data preprocessing of MARS model

The experiment was performed using the “R” software (version 4.1.2) (38) in R studio (version 1.1.453) (39); MLR was implemented with the “stats” package (version 4.1.2) (38); MARS was implemented with the “earth” package (version 5.3.1) (40); Lasso MLR was constructed by the “glmnet” package (version 4.1- 4) (41). For comparison purpose, classification and regression tree (CART) and random forest (RF) were also conducted. CART was conducted by the “rpart” package (version4.1.16) (42); and RF was built with the “ran-domForest” package(version 4.7-1.1) (43). In the modeling process, we randomly divided 80% of the dataset into a training dataset and the remaining 20% into a testing dataset. In the training dataset, a 10-fold cross-validation method was utilized for hyper-parameter tuning with the aid of the “caret” package (version 6.0-92) (44). When utilizing 10-fold cross-validation, the training set was randomly and equally divided into 10 folds (10% for each fold). Then, the 9-folds (90% of the training dataset) were used for training the model and the remaining 1-fold (10% of the training dataset) was used for validating the model. This process was repeated until each fold was used as validation once. Finally, after finding the best hyper-parameter set, the trained model used the testing data to evaluate the performance. The modeling process was repeated 10 times in our study, then we compared the results to determine the best-performing MARS model and get the equation from the selected one.

**Figure 2:**
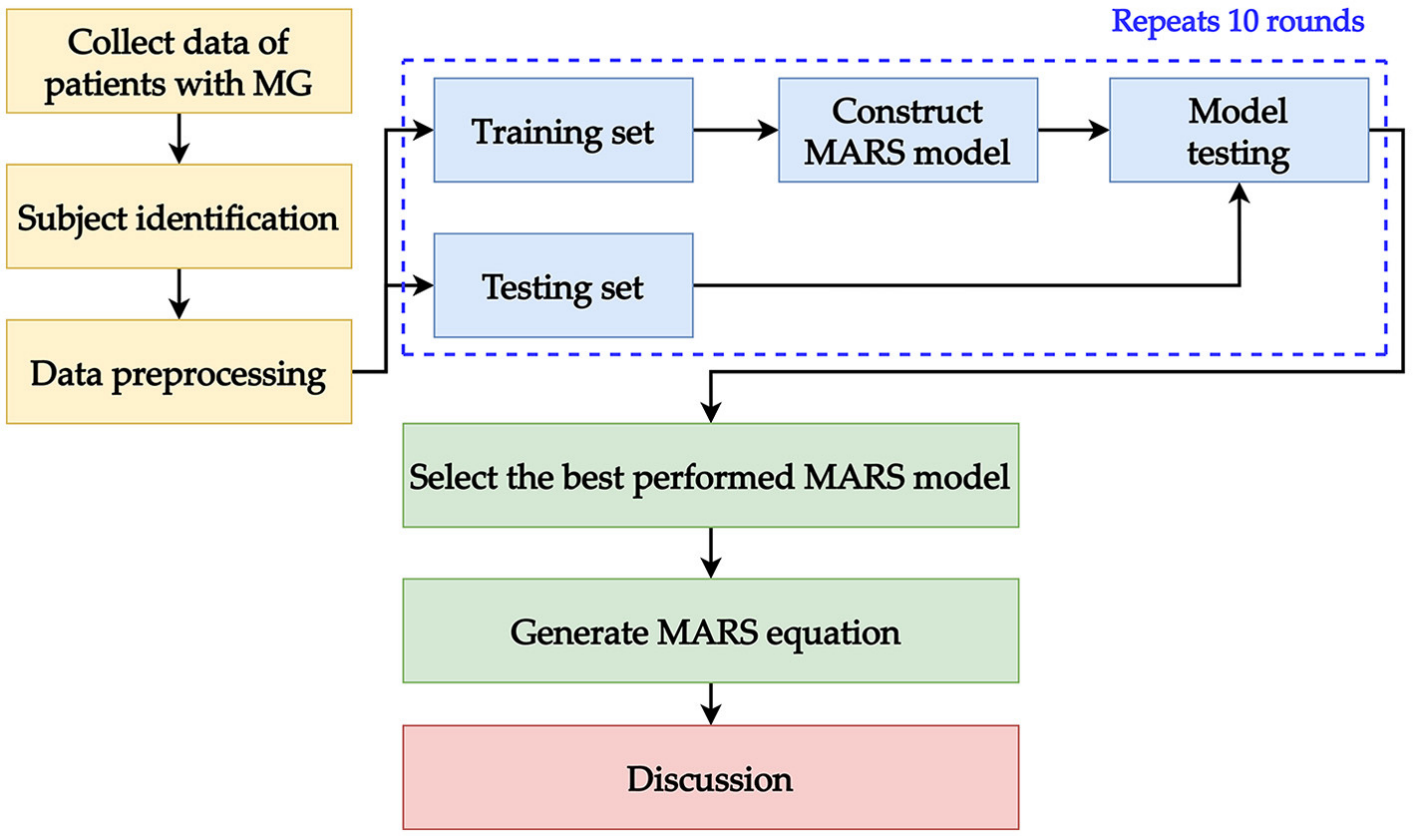
Data preprocessing processes for training and testing the MARS model.

For the performance evaluation, three metrics were used: mean absolute percentage error (MAPE), symmetric mean absolute percentage error (SMAPE), and relative absolute error (RAE). Using multiple metrics for evaluation ensures that the performance of the model is stable. These metrics measure the prediction error of model output and the model with the smallest error values is the best in terms of performance. MAPE and RAE were generated using the “MLmetrics” package (version 1.1.1) (45); SMAPE was generated using the “Metrics” package (version 0.1.4) (46). The described modeling process in this section is presented as a framework and shown in Figure 2.

## 3 Results

### 3.1 Characteristics

A total of 196 patients were included in the study, along with 19 clinical variables. The distribution of features in the entire dataset is listed in Table 1. The average age at admission was 49.4 years with women predominant (60.7%). The mean disease duration was 72.7 months. The average age at the onset of MG symptoms was 43-year-old. Among the patients, 88.8% displayed anti-AChR-antibody positivity and 4.1% showed anti-MuSK-antibody positivity. The average duration of hospital stay was 12.3 days. The MGFA clinical classification at admission divided the patients into several groups: 23 patients were classified as class I, 71 as class II, 64 as class III, 25 as class IV, and 13 as having an MG crisis with intubation. According to the thymus histology, 91 patients (46.4%) had thymoma and 59 patients (30.1%) had thymic hyperplasia. In total, 129 patients (65.8%) underwent thymectomy.

### 3.2 Performance of the MARS model

As mentioned, MARS is a non-parametric approach that can capture non-linear relationships between variables and can provide unique information. Nineteen predictor variables in this study were used when constructing the MARS model while three ML methods, namely, Lasso MLR, CART, and RF, were also constructed for comparison. Table 2 shows the model performance of the MARS model and the other three competing ML methods. According to the table, it can be found that the performance of MARS is similar with that of Lasso MLR, CART, and RF.

**Table 2:** Model performance of all four models used in this study.

**Methods**	**MAPE Mean (SD)**	**SMAPE Mean (SD)**	**RAE Mean (SD)**
MARS	0.524 (0.26)	0.409 (0.05)	1.133 (0.24)
Lasso MLR	0.526 (0.12)	0.401 (0.04)	1.123 (0.22)
CART	0.542 (0.16)	0.377 (0.04)	1.073 (0.28)
RF	0.521 (0.13)	0.370 (0.03)	1.007 (0.18)

To confirm the performance of the ML methods, the Kruskal–Wallis test (KW-test) and Wilcoxon signed-rank test (WS-test) were utilized to test the four methods. The WS-test is a non-parametric approach to the one-way ANOVA which can be used to compare multiple groups of data (47). The “KW-test” was first utilized for comparing MARS, Lasso MLR, CART, and RF. Then, to further check if the MARS generates a different model performance compared to the other three competing methods, the WS-test, a well-known non-parametric statistical technique to assess the prediction performance of two different algorithms (48), was used for pairwise comparison.

Table 3 shows the KW-test and WS-test results for comparing the performance of the MARS model to the Lasso MLR, CART, and RF models. From Table 3, it can be found that the MARS model does not have significant performance difference to the Lasso MLR, CART, and RF models. As mentioned in Section 2.2, the advantage and model characteristic of MARS are that it can capture the non-linear relationships between variables by assessing the knots and provide interpretable information through the knots from its equations, information which cannot be generated and provided by the Lasso MLR, CART, and RF models. Since the statistical testing results indicated that there is no significant performance difference among MARS and the three competing ML methods, the MARS model is the most suitable model of this study with extra helpful information to support clinical decision-making when predicting the average length of hospital stay for patients with MG.

**Table 3:** KW-test and WS-test results of the four used ML methods.

**ML methods**	***p*-Value**	**Significant**
**Kruskal–Wallis test**		
MARS vs. Lasso MLR vs CART vs. RF	0.7618	No
**Pairwise comparison of Wilcoxon signed rank test**		
MARS vs. Lasso MLR	0.437	No
MARS vs. CART	0.847	No
MARS vs. RF	0.766	No

### 3.3 Equation for prediction of length of hospital stay based on the MARS model

The BFs and coefficients of the best MARS model are listed in Table 4. As presented in the table, four important variables were selected by the best MARS, along with the corresponding knots, for which a total of seven BFs with seven knots were acquired from MARS. Based on Table 4, the MARS equation can be generated as follows:


$$\begin{array}{l} \text{ Average length of hospital stay}\\ =20.750-0.128\times BF1-0.013\times BF2-1.772\times BF3\\ +\;3.762\times BF4-1.180\times BF5-1.241\times BF6\;+\;1.268\times BF7 \end{array}$$

**Table 4:** Basis functions and important variables of the best MARS model.

**Corresponding equations of the model**		
	**Equation**	**Coefficients**
Intercept	—	20.750
**BFs**		
BF1	Max (0, Age at admission−41)	−0.128
BF2	Max (0, Disease duration−12)	−0.013
BF3	Max (0, 5–MGFA clinical classification)	−1.772
BF4	Max (0, MGFA clinical classification−5)	3.762
BF5	Max (0, 5–PSL maximum daily dose)	−1.180
BF6	Max (0, PSL maximum daily dose−10)	−1.241
BF7	Max (0, PSL maximum daily dose−15)	1.268

MGFA clinical classification stage I=1; IIa=2; IIb=3; IIIa=4; IIIb=5; Iva=6; IVb=7; V=8; The equation is the hinge function which takes the form of max (0, variable - knot) or max (0, knot - variable) (23).

### 3.4 Influence of the important variables

**Figure 3:**
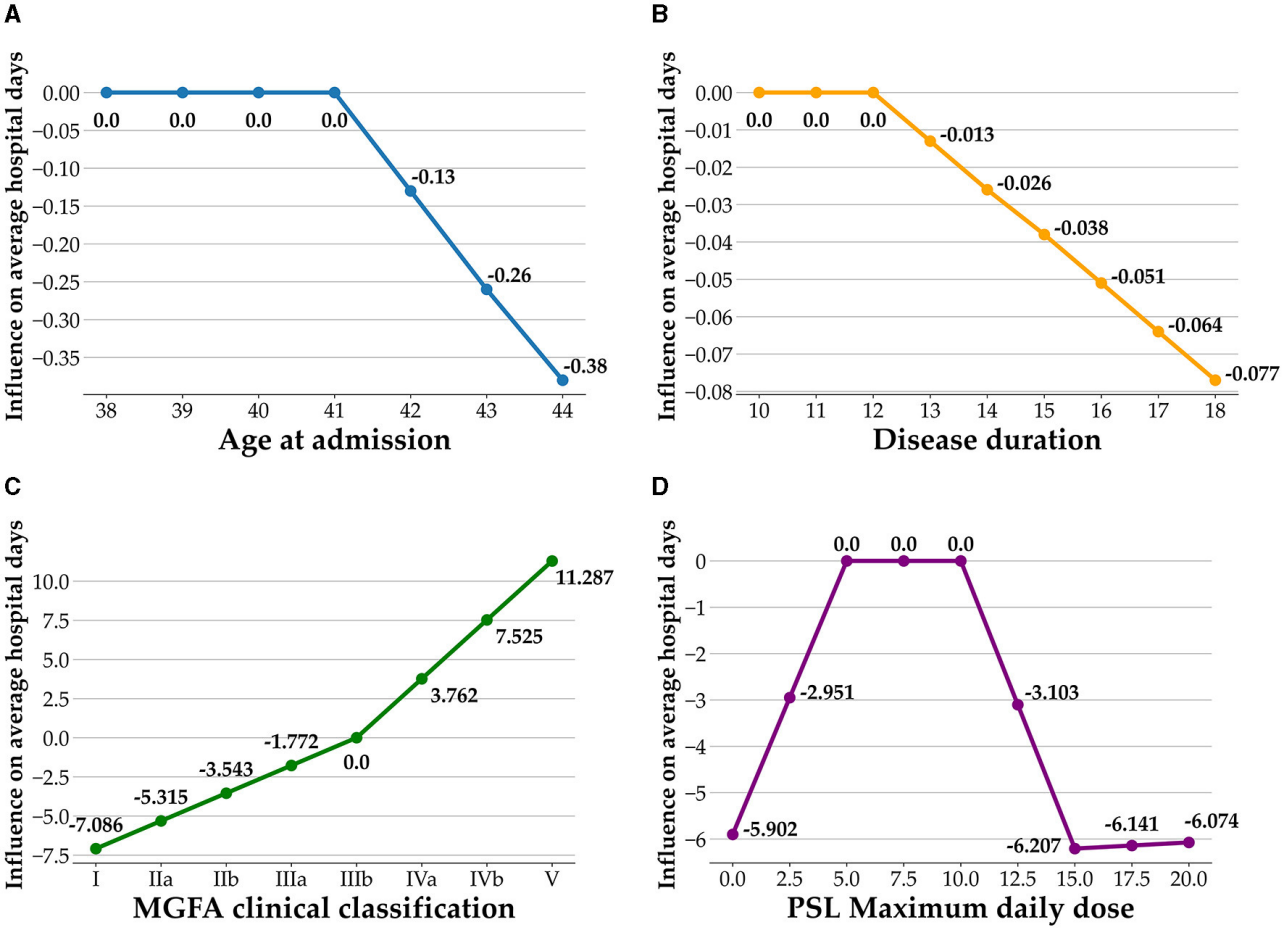
Influence of important variables on the average number of hospital days. **(A)** Age at admission; **(B)** disease duration; **(C)** MGFA clinical classification; **(D)** PSL maximum daily dose.

To better understand how the four important variables under the structure of BFs affect average length of hospital stay, Figure 3 presents a visualization of the influence of the important variables on the average number of hospital days. Each panel in the figure contains one of the important variables and corresponding BF. For example, the MGFA clinical classification has two BFs, which are plotted by combining the BFs and knots of the MGFA clinical classification. All of the panels in Figure 3 follow the same concept. In Figure 3, the influence of age at admission, disease duration, MGFA clinical classification, and maximum daily dose of Prednisolone (PSL) on average length of hospital stay are visualized. In Figure 3A, the age of 41 is the knot of variable age at admission; prior to age 41, there is no difference in the average length of hospital stay; after passing the age of 41, the average number of hospital days decreases. In Figure 3B, 12 months is the knot of the disease duration, and the average number of hospital days decreases after the duration exceeds 12 months. In Figure 3C, using MGFA clinical classification stage IIIb as the datum point, the average length of hospital stay is shortened when the MGFA clinical classification value decreases from stage IIIb. Further, when the values of MGFA clinical classification increase to stage IIIb, the length of the average length of hospital stay increases. Interestingly, in Figure 3D, the variable maximum daily dose of PSL has three knots, which are 5, 10, and 15 mg, respectively. When the maximum daily dose of PSL decreases from 5 mg, the length of average hospital stay shortens. The average length of hospital days remained no different when the maximum daily PSL dose was between 5 and 10 mg. A maximum daily dose of PSL between 10 and 15 mg shortened the length of the average length of hospital stay. Finally, when the PSL maximum daily dose increased from 15 mg, the average length of hospital stay increased.

## 4 Discussion

Using the data mining adaptive scheme with the MARS methodology, the study presented a result of four clinical variables that were important for the prediction of the average length of hospital stay, including age at admission, disease duration, MGFA clinical classification, and prednisolone dose, of which the non-linear relationships between them can be captured and described with the MARS equation. The MARS model demonstrated the cut-off point in the four factors and provided more detailed data on how these factors influence length of hospital stay. The data-mining based equation incorporating the four risk factors and detailed clinical parameters could provide good predictive accuracy in our sample.

It is important to assess the days in the hospital and risk factors that influence length of hospital stay at the time of patient admission because it is beneficial in terms of treatment protocols and financial plans for the hospitalization of MG patients (6, 31). In addition, it will be helpful for physicians to explain to patients, control risk, and make a decision plan that could improve the quality of care. MG is a rare disease; understanding the length of hospitalization is helpful for the formulation of national medical insurance policy (6, 31, 49). Several previous studies have tried to investigate the risk factors that influence the outcomes in MG with hospitalization based on tradition retrospective and regression analysis (6, 9, 31). One retrospective study demonstrated that intubation and PP correlated with hospital staying length and the male sex had correlation with a prolonged hospital stay (50). Respiratory distress and pneumonia had correlation with poor outcomes during hospitalization in a national-based review (31). The duration of corticosteroid administration can add to the burden of poor control MG (51). Our results showed another four clinical variables that relate to hospital stay length that could provide a direction and explainable result for evaluation of hospital stay for patients with MG.

There are some limitations of the traditional regression method for evaluation of the risk factors. First, if the clinical variables are non-linear or have collinearity, the strength of the relationship could be under-estimated. Second, it cannot determine the cut-off point between different factors. ML methods could address the weakness described above. Different machine learning techniques may need to be applied to various datasets (52). Our results also indicate that the MARS model was statistically significantly stronger than linear regression. Compared with MLR, MARS can automatically create a piecewise linear model that provides an intuitive stepping block into non-linearity after grasping the concept of multiple linear regression (23, 53). MARS is now a well-known ML method and has been used in some medical care issues, including optimal drug level detection, or applied in important variable cut-point detection (36, 54, 55).

Several previous studies have focused on the predictive factors for MG prognosis using data mining methods. A previous study using five ML methods showed that the MGFA classification, intravenous steroid administration during hospitalization, age, treatment with intravenous immunoglobulins, and thymoma were significant variables affecting prolonged hospitalization in MG patients (32). However, no studies have tried to assess length of hospital stay, and our study fills this gap. In contrast to previous results, which tried to identify the relatively important risk factors that related to prolonged hospital stay and the resulting target variable was a categorical variable (prolonged and non-prolonged), our results showed a good prediction accuracy of hospital stay length and the target variable is a continuous variable. We used the MARS methodology not only to identify the important risk factors that influence the average length of hospital stay but also to construct an easy-to-use model, and we can improve the model and prediction accuracy after incorporating this non-linearity.

Moderate MG symptoms at admission constituted the important variables in our datasets. The MGFA clinical classification is a standard method for identifying the different severities and clinical presentations of myasthenia gravis (56). The association of MGFA with length of hospital stay duration could be explained by the profound muscles weakness in these patients that cause severe disability. According to our results, there was a cut-off point at MGFA stage IIIb, as it is non-linear that the result of Figure 3 is not a straight line across the set of the findings. Furthermore, in the context of MG treatment, corticosteroids have been a first-line immunosuppressive therapy when symptoms are not adequately controlled (57). However, there is a possible risk of exacerbating MG, known as steroid-induced exacerbation, due to the mechanism involving lymphocyte depletion (58). The reported frequency of steroid-induced exacerbation varies (59), and the slow titration regimen is designed to reduce the risk (60). Thus, clinically, it is important to know what the best regimen is to avoid steroid-induced exacerbation and reach the optimal symptom control. However, currently there is no clear guidance. As a pilot study, our results showed the prednisolone dose had biphasic influence on hospital stay in patients with MG. It is possible that the higher doses of prednisolone for MG symptoms may cause prolonged hospitalization and provide an indicator of the impact of steroids on the length of hospital stay.

Several studies have emphasized the importance of age at onset as a prognostic factor for MG. A systematic review highlighted that an onset age below 40 years was a crucial factor for predicting remission (7). Johan et al. demonstrated that early-onset patients tend to have milder disease (40). Chinese studies indicated that MG patients with an onset age exceeding 40 years were more likely to develop generalized MG (41). Furthermore, a retrospective study found that elderly MG patients (onset age > 65 years) were prone to experience increased disease severity (42). Despite a higher percentage of patients in this subgroup presenting with life-threatening events and increase cost during admission, literature reviews have shown that elderly MG patients respond well to treatment (5, 6). While most studies traditionally focus on early/late-onset myasthenia gravis, typically distinguished by an age of 50, our research, although requiring further validation, has identified a critical age threshold at 41 years that influences prognosis. The use of the MARS methodology has introduced new variables and trends for assessing hospital stay duration. The precise impact of age on hospitalization remains unclear, necessitating further research for confirmation.

Our findings found disease duration is a factor that could influence the length of hospitalization. The association between the duration of the disease and the prognosis of MG has been a subject of controversy. Some reports have not concluded that disease duration is closely associated with prognosis in patients with MG (61). However, one cohort study identified disease duration exceeding 41 months as a factor negatively impacting the need for intensive care after MG admission (62). Additionally, a large retrospective study demonstrated that the risk of death tended to decrease after 15 years of the prevalence of the disease (63). Since it is an autoimmune disease, proper medical intervention helps stabilize the symptoms significantly (63). This may be due to the fact that a longer disease duration allows for more stable drug treatment and better psychological adaptation of the patient to the disease, resulting in a shorter length of hospitalization. Our findings, unlike those in other studies, have identified a specific threshold that a disease duration longer than 12 months negatively impacts the length of hospitalization. To the best of our knowledge, no prior study has established how the duration of the disease might affect MG outcomes. Our research offers fresh insights into the clinical care of MG.

The clinical implications of this study are that we constructed a MARS-derived model that can serve as a supportive assessment tool for clinical physicians in evaluating the length of hospital stay, which is rarely used in health care and allows us to model the interaction of explanatory variables. The interaction between the influencing factors found in this study has not been reported previously. After inputting the values for the four data points, a more accurate estimate of hospital stay duration in the clinician's diagnostic dataset can be derived, which can help in estimating medical costs and providing health education for patients with diseases. Moreover, because the variable phenotype of MG makes it difficult to determine the prognosis, physicians can use this model to identify patients likely to have prolonged hospitalization and the risk factors that influence it.

Despite these promising results, this study had some limitations. Firstly, the sample size was small, and it was drawn from a single center, which may reduce the generalizability of our results. Additionally, this model was not validated on a representative sample. For future validation and to enhance generalizability, data from multiple centers and various regions should be collected. Second, the data were collected from retrospective reviews, not prospective. As mentioned above, MG is a fluctuating disease; the MGFA classifies the disease according to the worst state the patient has been in and is not the best tool for grading patient severity at the time. It would have been better to use validated scales for MG such as the MG composite score (MGC) or the quantitative myasthenia gravis scoring (QMG), which can represent the disease severity and status, and also were not collected for analysis. In future studies, using prospective data for analysis can enhance model validation and improve the overall generalization and practicality of the ML model. Third, these models were chosen based on the clinical data. Other variables, such as blood samples, underlying comorbidities, and complications during hospitalization, were not included in our analysis. Incorporating this information could facilitate a more comprehensive analysis. Fourth, we excluded a significant number of cases from the original dataset because the admission reasons for these patients were unrelated to MG. This exclusion may affect the potential for future general applications. Fifth, our current study primarily focuses on the factors affecting the length of hospitalization after the admission. Therefore, we did not conduct an analysis of hospital stay duration based on different admission methods, including acute disease worsening leading to emergency admissions or admission to the intensive care unit. We also did not investigate the impact of hospitalization simply due to surgical procedures. Further studies should emphasize the impact of heterogeneity in hospitalization reasons, including factors like thymectomy surgery, as well as the ICU or emergency admission on the length of hospital stay. Finally, our study population were Asian, which significantly limits the generalizability of the study, and the pattern of clinical practice and admission criteria in this study may be different from those in other countries. Multicenter studies and increased sample size may complete the framework of this study to improve the performance of the MARS model and is worthy of further research.

## 5 Conclusions

Our results are the first to assess factors that influence the length of hospital stay using data mining methods. The result suggests that the ML-based models of hospital stay length in patients with MG should allow for non-linear associations that could improve their predictive ability. The non-linearity of the MARS model helped to identify cut-off points for four risk factors that influence hospital stay, including disease duration, age at admission, MGFA clinical classification, and daily dose of prednisolone. Furthermore, a MARS-based formula was designed as an assisting clinical decision support tool to help with the assessment of the average hospital stay in MG. In summary, the model maximizes predictions from measurements that can be feasibly supported as an extension of clinical risk assessments. The practical application of this model as a screening tool needs to be replicated and developed further, particularly in community settings.

## Data availability statement

The datasets presented in this article are not readily available because of ethical and privacy restrictions. Requests to access the datasets should be directed to C-CC, changcc75@gmail.com.

## Ethics statement

The studies involving humans were approved by the Research Ethics Review Committee of the Shin Kong Wu Ho-Su Memorial Hospital (No. 20190109R). The studies were conducted in accordance with the local legislation and institutional requirements. Written informed consent for participation was not required from the participants or the participants' legal guardians/next of kin in accordance with the national legislation and institutional requirements.

## Author contributions

C-CC: Conceptualization, Data curation, Formal analysis, Investigation, Project administration, Writing – original draft, Writing – review & editing. J-HY: Data curation, Writing – review & editing, Resources. H-CC: Data curation, Writing – review & editing, Resources. T-CL: Methodology, Software, Writing – original draft, Writing – review & editing. Y-MC: Data curation, Writing – review & editing, Resources. M-JJ: Methodology, Software, Writing – original draft. C-JL: Conceptualization, Methodology, Project administration, Supervision, Writing – original draft, Writing – review & editing, Formal analysis, Investigation, Funding acquisition.
